# Crosstalk between the microbiota and intestinal dendritic cells in IBD

**DOI:** 10.1007/s00281-025-01062-9

**Published:** 2025-09-30

**Authors:** Philine Letz, Samuel Huber, Lis N. Velasquez

**Affiliations:** 1 Department of Medicine, University Medical Center Hamburg-Eppendorf, Hamburg, Germany; 2 Hamburg Center for Translational Immunology (HCTI), University Medical Center Hamburg-Eppendorf, Hamburg, Germany

**Keywords:** Dendritic cells, Microbiota, IBD, Crohn’s disease, Ulcerative colitis

## Abstract

Conventional dendritic cells (cDCs) play a pivotal role in orchestrating the delicate balance between immunity and tolerance within the gastrointestinal tract by interacting with other cell types, particularly T cells. Meanwhile, the microbiota is critical for the induction and modulation of the immune system in the gut and plays a key role in the function of cDCs. So far, the study of intestinal cDCs has been encumbered by their limited numbers and phenotypic overlap with other myeloid cells. Recent advancements in single-cell sequencing technology have helped define cDCs and their subsets, while also providing valuable insights into the contribution of cDCs to Inflammatory Bowel Disease (IBD). However, the exact role of cDCs in IBD remains unclear, particularly in terms of how the microbiota influences their function in this context. In this review, we summarize the functions of cDCs in the intestine and during IBD, and the role of the microbiota in cDC biology. We also describe the current limitations in the study of cDCs and the microbiota, as well as new methods for studying DC-T cell communications in vivo, which can help increase our understanding of the function of cDCs in the intestine and develop new therapeutic strategies against IBD.

## Introduction

Inflammatory bowel disease (IBD) is a chronic inflammatory disorder with increasing incidence in developed and developing countries. Despite its unknown etiology, genetic factors, environmental influences, changes in the intestinal microbiota composition, and dietary habits were shown to play a role in the development of the disease [1]. IBD can be subclassified into two major forms, ulcerative colitis (UC) and Crohn’s disease (CD). Whereas UC is mainly characterized by mucosal inflammation spanning the rectum and the lower gastrointestinal tract (GIT) in a continuous manner, CD affects the whole GIT with patchy lesions interposed by uninflamed tissue [2, 3].

In recent years, sophisticated analysis methods, such as single-cell sequencing and spatial transcriptomics, have emerged that allow for the examination of the intestinal cellular and molecular landscape in IBD at increased resolution. In contrast to the T cell-oriented focus of IBD research in the past decades, these analyses shed light on the heterogeneous cellular composition present in IBD lesions and suggest that other immune cell types, such as myeloid cells, are also critical in the pathogenesis of IBD and are even involved in therapy resistance [4–6]. In line with this, genome-wide association studies (GWAS) have identified multiple risk genes for IBD, many of them expressed by dendritic cells and macrophages, involved in host-microbe interactions, or related to the function and migration of these cells [7, 8]. Not only did the host immune cell composition get disentangled at a higher resolution, but also the microbial communities in the intestine. The complex relationship between host and the microbiota, consisting of bacteria, as well as Archaea and Eukarya, is mostly symbiotic. However, in several autoimmune diseases, such as IBD, a dysbiosis of the delicate composition of commensals is commonly observed [9]. Yet its influence on myeloid cells and whether it can be causally involved in IBD pathogenesis remains to be fully elucidated. Furthermore, recent analysis revealed that in addition to the marked differences between the microbiota of people with IBD and healthy individuals, the gut microbiota is subject to inter-patient variation and longitudinal changes reflecting alterations in disease severity and activity [10, 11]. To date, little is known about how these physiological modifications of the intestinal microbiota affect the function of the various immune cells involved in IBD pathogenesis.

Although other subsets of myeloid cells, such as monocytes/macrophages, play a significant role in IBD, we have decided to focus on conventional dendritic cells (cDCs) here. As they bridge the innate and adaptive immunity by their ability to sample, process, and present antigens to T cells, thereby inducing pathogenic responses or ensuring the maintenance of tolerance, cDCs play a pivotal role in the interplay of microbiota and host immunity.

In this review, we will describe the various cDC subsets, their primary role in the gut, and their contribution to IBD. Next, we will discuss how the microbiota influences the function and phenotype of cDCs, taking into account the changes occurring along the GIT. Finally, we will examine the effect of the microbiota on DC-T cell interactions and discuss new methodologies for studying this in vivo.

## Intestinal dendritic cells and their role in IBD

### Dendritic cell subsets in the intestine

**Fig. 1:**
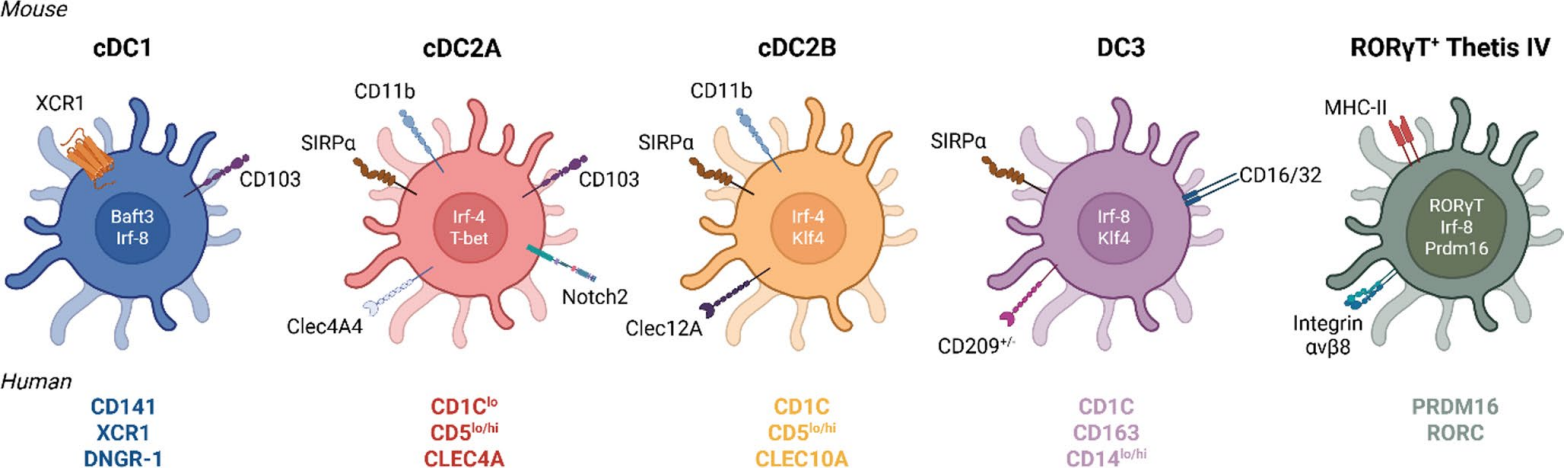
Mouse and human dendritic cell subsets

Intestinal cDCs can be found in the lamina propria as well as other induction sites such as the Peyer´s Patches and isolated lymphoid follicles (ILFs) throughout the gastrointestinal tract [12]. Distinct subsets of DCs have been described in the literature; here we will focus on conventional DCs (cDCs), excluding monocyte-derived subsets and plasmacytoid DCs (pDCs), which have been described elsewhere [13, 14]. Lately, the field has benefited from the refinement of single-cell transcriptomic techniques and the development of novel ontogeny-tracking mouse models, which have enabled us to define the markers and transcription factors that characterize each cDC subset in both mice and humans with greater accuracy. As a result, we can distinguish two main cDC subtypes that differ in their phenotypic markers and functions: cDC1s and cDC2s (Fig. 1).

cDC1s are defined by the expression of the chemokine receptor Xcr1 and the surface markers CD103 or CD8α. This subset depends on the transcriptional regulation of Baft3 and Irf8 for their differentiation and is proposed to be highly efficient in cross-presenting antigens and promoting type 1 immune responses. On the other hand, cDC2s require Irf4 for their development and migration capacity, and they express high levels of the surface markers CD11b and SIRP-α. cDC2s also preferentially interact with and promote CD4^+^ T cell-mediated responses [15].

cDC2s are a more heterogeneous population and are subdivided into two subtypes, cDC2A and cDC2B, depending on whether their differentiation is driven either by the Notch2 receptor or the Klf4 transcription factor, respectively. These subtypes can be primarily distinguished by the expression of T-bet as well as other markers, which vary according to the organ where the cells come from [16, 17]. cDC2As are T-bet^+^ and express CD4, Clec4A4, and Esam in the spleen and CD103 in the intestine [16, 18], whereas cDC2Bs are T-bet^−^ and express Sirp-α and Clec12A [17, 19, 20]. The transcriptional divergence of these subsets was recently found to appear in the pre-DC2 stage. Specific progenitors can be found in the bone marrow which give rise to the cDC2A and cDC2B subsets in a Tcf4- and Klf4-manner respectively [17, 21].

Other newly identified DC subsets correspond to DC3s and RORγT^+^ APCs. Murine DC3s are characterized as Sirp-α^+^ CD16/32^+^ and arise from distinct Ly-6 C^+^ monocyte-DC progenitors (MDPs). They have been shown to have superior Th17-priming capacities compared to cDC2s though their function in vivo is still unclear [22]. RORγT^+^ APCs are a recently described constellation of cells defined by their expression of MHC-II and the transcription factor RORγT. Though they received different names, they seem to correspond to a new cell type termed Tethis cells (TC), which share transcriptional features of both medullary thymic epithelial cells (mTECs) and DCs [23, 24]. From the four subsets identified (TC I-IV), TC IV has been implicated in the early differentiation of peripherally-induced Tregs (pTregs) against food and microbial antigens (Ags) [23–30].

Human dendritic cells can also be divided into cDC1s, cDC2s, and DC3s (Fig. 1). Human cDC1s are characterized by the expression of BDCA-3 (CD141), XCR1, and DNGR-1. On the contrary, cDC2s and DC3s share the expression of BDCA1 (CD1c) but can be subdivided into CD5^+^ cDC2s and CD163^+^ DC3s [31–33]. Comparisons between human and murine DCs populations have aligned cDC2s and DC3s to the to the mouse cDC2A and cDC2B subpopulations respectively [16, 22]. However, the discovery of distinct cDC2A and cDC2B precursors in mice indicates that human CD1c^+^ subpopulations might be more heterogenous than thought and their nomenclature needs to be revisited.

Overall, the DC family is now expanding, though the question remains whether these new subsets can be considered cDCs or they correspond to other myeloid compartments. A defining criteria for cDCs is their ability to activate naive T cells. Thus, future studies are needed to determine this as well as the existence of tissue-specific subpopulations and their exact functions.

Conventional dendritic cells (cDCs) can be divided into two subtypes, cDC1s and cDC2s. In addition, two types of cDC2s can be distinguished: cDC2As and cDC2Bs. Newly identified DC subsets include DC3s and RORγT^**+**^ APCs. The defining markers to distinguish each subset and the transcription factors driving their differentiation are shown. Below each population, the respective markers for each subpopulation in humans are shown.

## Functions in the intestine

**Fig. 2:**
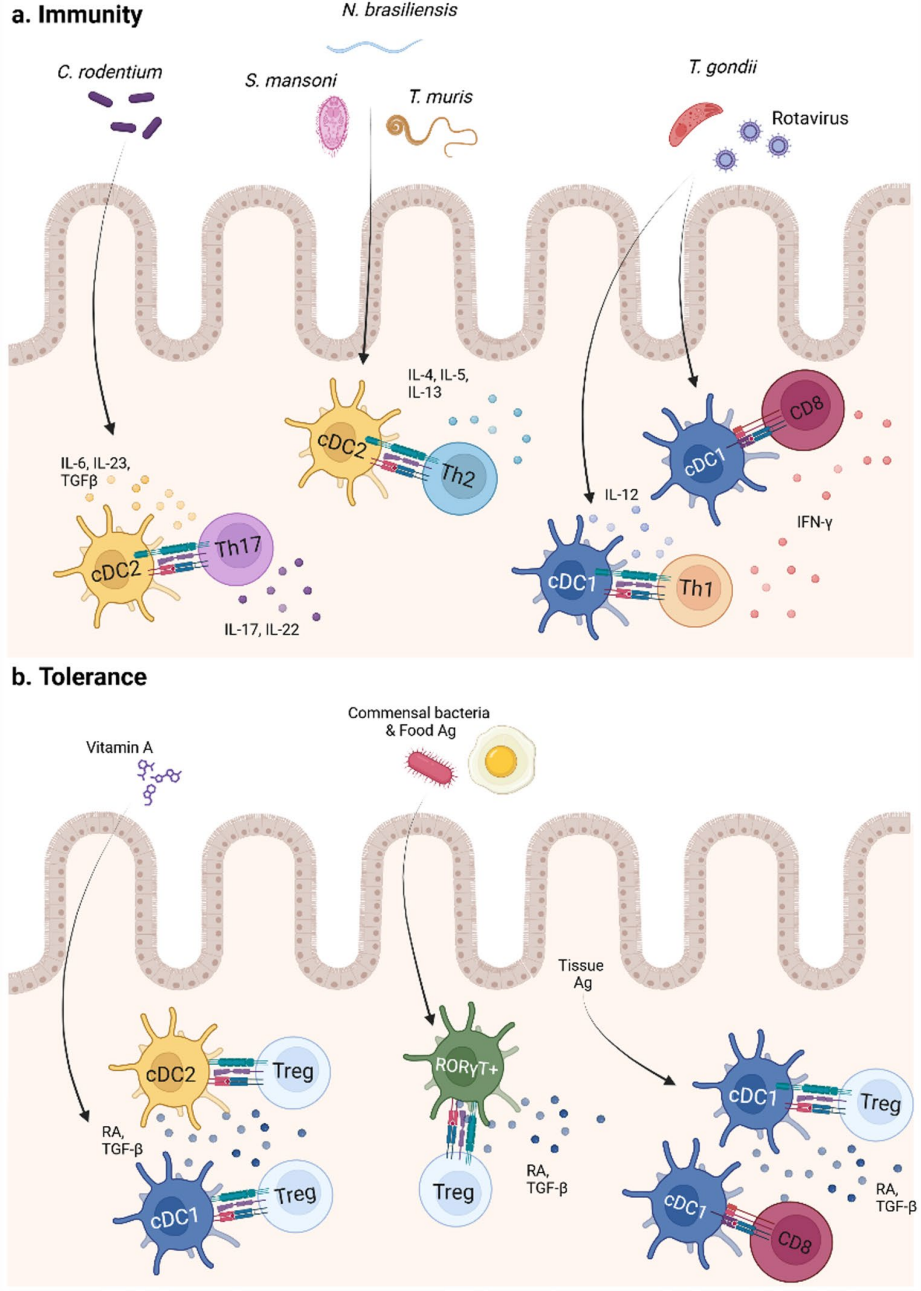
Functions of intestinal cDCs

The most prominent functions of cDCs derive from their ability to interact and activate naïve T cells. As a result of this interplay, cDCs are proposed to play crucial roles in initiating potent adaptive immune responses, while at the same time regulating tolerance (Fig. 2). Each cDC subset has been shown to preferentially interact with a different T cell subtype, thereby promoting distinct types of immune responses. Therefore, cDC1s excel at cross-presenting Ags to CD8^+^ T cells and are involved in promoting type 1 immune responses, whereas cDC2s are associated with the activation and initiation of different CD4^+^ T cell programs [34]. Several studies employing cell ablation models and conditional knock-out (cKO) mice for the different DC subtypes support this concept. Below, we detail the main functions of cDCs, many of which were described utilizing gut pathogens or pathobionts.

Even though there is no strong evidence suggesting that pathogens cause IBD, many of the genetic variants associated with increased risk for IBD correlate with an impairment in pathogen recognition. This suggests that a weakened barrier function in the gut, possibly facilitating the growth of pathogenic species, could eventually promote IBD [35].

Depletion of cDC1s, either by targeting Irf8 in CD11c^+^ cells or by crossing Xcr1^cre^ to diphtheria toxin A subunit (DTA) mice, results in an imbalance between Th1 and Th17 populations at steady state in the small intestine (SI) and colon. Mice lacking cDC1s have fewer IFN-γ-producing CD4^+^ T cells and a concomitant increase of IL-17 A^+^ CD4^+^ cells [36, 37]. Mechanistically, the accumulation of homeostatic intestinal Th1 cells was shown to rely on cDC1-dependent Ag presentation and IL-27 secretion [38]. The ability of cDC1s to favor the differentiation of type 1 responses was also shown in the context of pathogenic viral and parasitic infections. Indeed, lack of Baft3-dependent cDC1s results in a reduction of rotavirus (RV)-specific CD8^+^ T cells, reduced IFN-γ^+^ CD4^+^ T cells in neonate mice, and RV-specific IgA production, ensuing prolonged viral shedding into the gut lumen [39, 40]. Baft3^KO^ mice also succumb to infection with the intracellular parasite *Toxoplasma gondii*, showing reduced IL-12 and IFN-γ secretion post-infection and a defect in priming specific CD8^+^ T cells [41].

Conversely, both cDC2As and cDC2Bs have been implicated in promoting CD4^+^ T cell immunity. Results derived from single-cell sequencing data, corroborated in vitro, suggest that both cDC2 subtypes have a similar capacity to induce CD4^+^ T cell proliferation, but that cDC2Bs are more pro-inflammatory and superior at promoting the differentiation of Th17 cells [16, 22]. However, in vivo, both cDC2 subtypes were shown to be able to direct Th17 responses. Indeed, deletion of Notch2, Irf4, and Tgfbr1 in CD11c^+^ cells results in the absence of cDC2As in the SI and a parallel reduction in homeostatic IL-17 A-producing CD4^+^ T cell numbers, possibly because of a decline in cDC2A-dependent expression of IL-23, IL-6, and TGF-β [18, 42–44]. Likewise, the lack of Notch2-dependent cDC2As renders mice susceptible to *Citrobacter rodentium*, suggesting that this subset is critical to promote Th17 immunity against bacterial pathogens by secreting IL-23 and regulating IL-22-producing ILCs [45]. Reduced numbers of IL-17 A^+^ CD4^+^ T cells in the skin were also observed in cDC2B-deficient Klf4^fl/fl^ mice crossed to CD11c^cre^, confirming their contribution to Th17 differentiation too [20]. Additionally, cDC2s can also support Th2 responses in the context of intestinal helminth infections. Mice deficient in Irf4 in CD11c^+^ cells secrete less IL-4, IL-5, and IL-13 both in the SI and colon after infection with the parasite *Schistosoma mansoni*, and a similar outcome is observed after infection with *T. muris* and *Nippostrongylus brasiliensis* [46–48]. Analogous results are also available for cDC2Bs, where Klf4^cKO^ mice show increased susceptibility to *S. mansoni* infection and a defect in inducing a Th2 immune response [19].

The second main function of cDCs is related to their ability to preserve intestinal tolerance. In this regard, both cDC1s and cDC2s were shown to be necessary to drive Treg responses after oral/rectal administration of the model Ag OVA, though cDC1s and cDC2As seem to have preferential pTreg-polarizing abilities [44, 49, 50]. Likewise, the generation of specific pTregs against species of the gut pathobiont family *Helicobacter* relies on multiple cDC subsets [51]. Thus, it is possible that different cDC populations can promote pTreg differentiation, but this depends on the cellular and tissue context. cDCs can favor the generation of pTregs via their enhanced expression of RALDH2 (encoded by Aldh1a2), which catalyzes the synthesis of retinoic acid (RA), as well as TGF-β and the TGF-β-activating integrin αvβ8 [52–54]. However, whether all cDCs rely on the same methods is unclear, as most of these mechanisms have been proven in vitro with cells isolated from the SI. Indeed, though most studies point towards a critical role of TGF-β and its signaling pathway, other cDC-produced cytokines such as TSLP and IL-33 also support the expansion of Tregs in the intestine [55–58]. cDCs have also been proposed to orchestrate tolerance to tissue-derived Ags, though in this case, cDC1s seem to be the primary subset involved due to their enhanced ability in cross-presenting Ags. Thus, cDC1s were shown to be able to cross-present intestinal epithelial cells (IECs)-derived OVA to CD8^+^ T cells and, via a mechanism involving PD-L1, TGF-β, and RA, induce the differentiation of specific Foxp3^+^ CD8^+^ Tregs that prevent intestinal inflammation and epithelial destruction [59, 60]. However, induction of regulatory T cells is not the only mechanism, since the absence of MHC-II on cDC1s can also exacerbate the cross-presentation of keratinocyte-derived OVA, which triggers the expansion of self-reactive CD8^+^ T cells, leading to fatal multi-organ autoimmunity [61].

The role of cDCs in the differentiation of pTregs has been lately challenged by the identification of RORγT^+^ APCs. MHC-II^∆RORγT^ and Rorc_E_+7kb^∆/∆^ mice, which either show impaired Ag presentation or are depleted from this cell type altogether, develop gut inflammation and a concomitant loss of pTregs against commensal bacteria and food Ags. Utilizing single cell sequencing techniques and fate-mapping reporter mice, the RORγT^+^ TC IV subset was identified as the population implicated in the early induction of pTregs via the expression of the TGF-β-activating integrin αvβ8 and Aldh1a2 [23–30]. Since RORγT^+^ APCs express the canonical cDC markers CD11c and Zbtb46 at some point in their development, they may have been overlooked in previous studies focusing on the differentiation of pTregs [28]. However, though recent evidence suggests that cDCs are irrelevant for the induction of pTregs, they seem to collaborate with the RORγT^+^ TC IV subset in maintaining them after their differentiation [24, 30]. Thus, cDCs preserve intestinal tolerance, most likely via the mechanisms described above, despite not being involved in the first wave of pTreg differentiation. Other unanswered questions regarding the role of RORγT^+^ APCs in pTreg promotion remain, such as whether RORγT^+^ APCs are present in all intestinal locations and whether they can also induce pTregs later in the lifecycle, functions which could be then adopted by cDCs.

To conclude, each cDC subset has specified functions that are well established. However, new data suggest they can have some degree of overlap in their functions. Yet, the local conditions that dictate which subset is preferred over the others in each circumstance are unknown.

Conventional dendritic cells (cDCs) play a crucial role in inducing adaptive immune responses and promoting tolerance in the intestine. (a) cDC1s excel at cross-presenting antigens (Ags) and can induce the production of IFN-γ by CD8^+^ and Th1 cells against viruses such as rotavirus (RV) and intracellular pathogens like *T. gondii*. In contrast, cDC2s can recognize extracellular bacteria such as *C. rodentium* and promote Th17 cells that secrete IL-17 A and IL-22 via the production of IL-6, TGF-β, and IL-23. Additionally, cDC2s are critical to induce IL-4, IL-5, and IL-13 in Th2 cells and aid in the protection against helminth pathogens such as *N. brasiliensis*, *S. mansoni* and *T. muris.* (b) Both cDC1s, cDC2s, and the newly identified subset of RORγT^+^ DCs are implicated in driving responses of regulatory T cells (Tregs) against food and bacterial antigens (Ags) via the production of retinoic acid (RA) and TGF-β. Furthermore, cDC1 can cross-present tissue Ags to CD8^+^ T cells and promote tolerance by the induction of Tregs.

## Functions in IBD

One of the most common inflammatory diseases affecting the gastrointestinal tract is IBD. The exact contribution of cDCs to this disease is still largely unclear. However, evidence suggests that cDCs play a critical role in both the development and progression of IBD. Susceptibility to IBD is known to be hereditary, and several loci have been identified via GWAS, which confer increased risk of developing the disease [7, 8]. Many of these loci are expressed by cDCs, indicating that genetic defects in these cells, particularly regarding pathogen recognition, are essential for maintaining homeostasis in the gut and preventing inflammation. Examples of these genes are NOD2, TNFAIP3, and XIAP. IBD-associated NOD2 and ATG16L1 variants can impair the ability of DCs to undergo autophagy and present Ags to CD4^+^ T cells [62]. Additionally, DCs isolated from people with Crohn’s disease carrying NOD2 variants associated with susceptibility to IBD show reduced NOD2-dependent expression of miRNA-29, which exacerbates the production of IL-12p40 after exposure to invasive adherent *E. coli* [63]. Similarly, conditional deletion of A20, a suppressor of pro-inflammatory signaling pathways encoded by Tnfaip3, in CD11c^+^ cells, leads to early spontaneous colitis development in the SI and delayed colon inflammation after 6 months of age [64, 65]. Lack of A20 expression results in cDCs with a mature phenotype, which promote the expansion of Th1 and Th17 cells that drive the pathology [65]. Finally, a deficiency in XIAP causes severe IBD in about one-third of individuals carrying this mutation. XIAP^−/−^ mouse models suggest tissue damage results from malfunction and loss of myeloid cells, potentially due to TNFR2-mediated cell death following TLR5 activation. Human IBD patient single-cell sequencing reveals a cDC population with high expression of TLR5 and TNFR2, absent in healthy individuals, indicating that TLR and TNF signaling in cDCs may play a role in IBD pathogenesis [66].

In line with this, multiple studies analyzing single-cell sequencing analyses of IBD lesions have shown that myeloid cells have a hyperinflammatory state and express genes associated with therapy resistance [4–6]. In therapy-refractory individuals with ileal Crohn’s disease, Martin et al. identified a cellular module called GITMAS, which comprises hyperinflammatory cells, including activated cDCs. The cellular structure of the GITMAS module seems to depend on a specific cytokine-chemokine network, with activated DCs substantially contributing to the recruitment of activated T cells and cytotoxic T lymphocytes (CTLs). Consequently, activated DCs may have a detrimental impact on the initial steps of lymphocyte aggregate formation in the intestinal lamina propria, contributing to the failure to achieve remission under α-TNF therapy [4]. This correlates with flow cytometry studies suggesting that lamina propria DCs from people with IBD express higher levels of Toll-like receptors (TLRs) and secrete higher amounts of pro-inflammatory cytokines compared to controls [67–69]. However, whether this hyperinflammatory phenotype observed in IBD is a cause or a consequence of the disease has not been elucidated. Furthermore, cDCs were also found to have underestimated effects on the mechanism of action of Vedolizumab. The integrin α4β7 blocker mainly works by preventing CD1c^+^ DCs from migrating to the intestinal mucosa, opposing the previous notion that Vedolizumab primarily inhibits CD4^+^ memory T cells from migrating to the intestine. The findings emphasize the significant role of cDCs in the pathogenesis of IBD [70].

Correlating to the findings in humans, murine models of intestinal inflammation show that cDCs in IBD can have a beneficial role by limiting the inflammation, or they can themselves gain altered, detrimental functions and further promote the disease. For example, colonic cDCs are a primary source of IL-22 binding protein (IL-22BP) during homeostasis and IBD. During the recovery phase after intestinal tissue damage, cDC-derived IL-22BP can neutralize and control the tissue repair functions of IL-22, which, if left uncontrolled, can exacerbate the inflammation and eventually lead to tumorigenesis [71–73]. The loss of IL-10 signaling in cDCs causes spontaneous inflammation in the SI, characterized by lymphocyte infiltration into the epithelium and lamina propria, as well as an overactivated phenotype of the cDCs with increased production of proinflammatory cytokines, including IL-6 and TNF-α. Although IL-10 signaling in T cells remained unaffected, increased secretion of IFN-γ and IL-17 A by T cells exacerbated inflammation in this model. This implies that the regulation of IL-10 signaling in intestinal cDCs is necessary to control T cell responses, challenging the assumption that IL-10 signaling in T cells alone is sufficient to limit inflammatory T cell responses [74]. In addition, cDCs contribute to maintenance of homeostasis by their ability to induce pTregs against various Ags. Accordingly, the loss of this function or changes in the cDC subsets can affect Tregs numbers in the gut and lead to colitis development [44, 56]. In the TNFΔARE ileitis model, which resembles the inflammation present in CD, CX3CR1^+^ mononuclear phagocytes accumulate in the inflamed regions, accompanied by a decrease in pro-tolerogenic CD103^+^ DCs [75]. However, treatment of the animals with Flt3-L could induce an increase in CD103^+^ DCs, and consequently, upregulation of RALDH2, which leads to the subsequent release of RA by cDCs. This results in an increase in Tregs, as well as a decrease in the severity of inflammation. Thus, cDCs can control intestinal inflammation. However, whether they lose these functions in IBD or become insufficient to control other inflammatory cells is yet to be defined.

Shifts in the function of cDCs, which lead to the suppression or development of new cDC-dependent immunological mechanisms in the intestine, have also been implicated in the development of IBD in mice. Clec9A^DTR^ mice, which lack cDC1s, have increased susceptibility to DSS-induced colitis related to diminished induction of IFN-γ-induced genes (ISGs) in IECs, suggesting that the interplay between cDC1s and IECs is impaired, and upon barrier disruption, IECs cannot respond appropriately to invading commensals [76]. In general, alterations in cDC function seem to promote aberrant interactions with other cells. In this context, a complex interplay between T follicular helper cells (Tfh) and cDCs can contribute to the development of colitis. DCs were shown to enhance Tfh differentiation in colonic lymphoid follicles, while at the same time, Tfhs promote the recruitment of mature DCs and the formation of DC-T cell clusters, further favoring the expansion of Tfhs. Once in the lamina propria, Tfhs acquire a Th1 phenotype and can contribute to the outbreak of colitis [77].

Another relevant question is which cDC subset is mainly affected in IBD. Analysis of IBD lesions at the single-cell level uncovered that mainly cDC2 gene expression seems to be altered in IBD, corresponding with a hyperinflammatory phenotype [4–6]. However, subset ablation experiments do not unequivocally confirm this and present different outcomes, depending on the targeting genes used and the model of colitis (Table 1). In favor of a predominant aberrant function of cDC2s in IBD, Clec4a^DTR^ mice are protected against DSS-induced colitis and have increased epithelial barrier integrity [76]. Similarly, Rag1^KO^ Irf4^fl/fl^ CD11c^cre^ animals show a delay in the onset of T cell-induced colitis, though they present no differences compared to control animals at later stages of the disease [78]. On the contrary, Rag1^KO^ Tgfbr1^fl/fl^ CD11c^cre^ mice develop severe colitis after T cell transfer, instead suggesting that certain cDC2 functions could also contribute to preventing IBD development [44]. Regarding cDC1s, their depletion using Xcr1^DTA^ and Clec9A^DTR^ mice results in increased susceptibility to DSS-induced colitis [37, 76]. However, Baft3^cKO^ shows no increased sensitivity to the same colitis model [79].

In summary, cDCs in IBD predominantly exhibit a hyperinflammatory phenotype and aberrant functions, possibly because they carry gene variants that impair their pathogen-recognition or Ag-processing abilities and promote the pathogenesis of IBD. However, several questions, such as the subtype of cDC and the molecular mechanisms involved, remain unanswered. Furthermore, the role of the intestinal landscape, particularly the local microenvironment, has not been studied in detail so far. This could explain some of the inconsistencies regarding cDC contributions in IBD.

**Table 1:** Effects of genetic modifications in cDCs on colitis models

Mainly affected cell type	Genetic modification	Colitis model used	Effects
cDC1	***XCR1*** ^**DTA**^	DSS colitis	Increased susceptibility to DSS colitis [37]
	***Clec9a*** ^**DTR**^	DSS colitis	Diminished induction of IFNγ-induced genes in IECs, impaired interplay of cDCs and IECs [76]
	***Baft3*** ^**cKO**^	DSS colitis	No increase in DSS severity [79]
cDC2	***Clec4a*** ^**DTR**^	DSS colitis	Protection against DSS colitis, improved barrier integrity [76]
	***IRF4***^**fl/fl**^ **CD11c**^**cre**^	T cell transfer colitis	Delay in onset, no differences in severity at later stages of disease [78]
	***Tgfbr1***^**fl/fl**^ **CD11c**^**cre**^	T cell transfer colitis	Severe colitis development after T cell transfer [44]
cDC	***A20***^**fl/fl**^ **CD11c**^**cre**^	DSS colitis; T cell transfer colitis	A20-deficient mice developed spontaneous colon inflammation; Promotion of Th17 and Th1 expansion; Exaggerated weight loss in DSS- and T cell transfer colitis [64, 65]
	***Il10ra***^**fl/fl**^ **CD11c**^**cre**^	Spontaneous inflammation	Development of small intestinal inflammation; Increased production of proinflammatory cytokines by cDCs and expansion of IFNγ- and IL-17 A-producing T cells [74]

## Influence of the microbiota on DCs

### Influence of the microbiota via pattern recognition receptors (PRR)-signaling

**Fig. 3:**
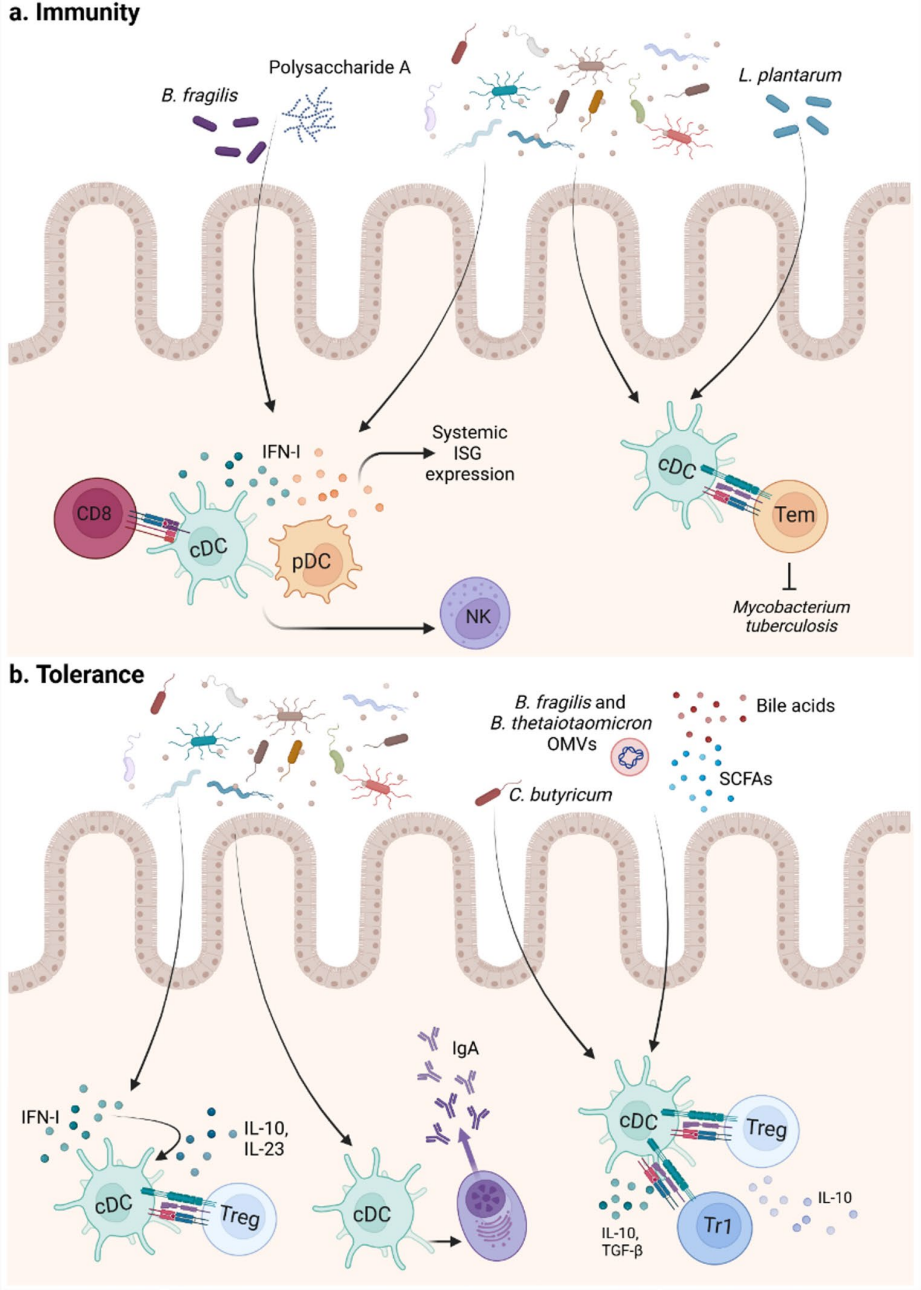
Influence of the microbiota and their metabolites on the function of cDCs

cDCs play two vital roles, one orchestrating immunity against pathogens and the other promoting intestinal tolerance. The role of the microbiota was previously thought to be passive, only involving recognition by cDCs. As a result, cDCs would then induce the differentiation of pTregs to dampen unwanted inflammation. However, studies in germ-free mice showed that the complete absence of commensals drastically impairs the development of the intestinal immune system. Consequently, this demonstrated that the microbiota plays an active role in the functional maturation of immune cells in several aspects [80]. The microbiota was also shown to act in two major ways: on the one hand, it primes the immune system to respond to pathogens, on the other hand, microbial influences promote the induction of tolerance (Fig. 3). Among the mechanisms employed to instruct cDCs on how to combat pathogenic species, it was recently described that the commensal microbiota can control type I IFN (IFN-I) production by plasmacytoid DCs (pDCs) and colonic DCs. Sensing of IFN-I impacts the steady-state functions of cDCs and their ability to respond to subsequent microbial and viral stimulation. In both cases, the microbiota is detected via PRRs, either collectively as in the case of pDCs or by specific recognition of *Bacteroides fragilis* polysaccharide A (PSA) via TLR4 in colonic DCs, which promotes the induction of IFN-I and upregulation of ISGs [81, 82]. By signaling via IFNAR, microbiota-induced IFN-I primes cDCs for functional reactions to viral pathogens, promoting antiviral NK and T cell reactions. Likewise, dysbiosis induced by broad-spectrum antibiotics abrogated Mincle expression, a PRR from the C-type lectin family, in lung DCs. These reduced Mincle levels impaired the response and phenotype of lung DCs, resulting in defective T cell responses and increased *Mycobacterium tuberculosis* (*Mtb*) survival. However, a proficient immune response against *Mtb* after antibiotic treatment could be restored by supplementation with the commensal *Lactobacillus* or a synthetic Mincle agonist [83]. Thus, the microbiota can instruct cDCs on how to mount a proper immune response against bacterial and viral pathogens, whereby IFN-I seem to be key cytokines involved in this process.

cDCs are also of fundamental importance for the induction of tolerance in the intestine. In this regard, the microbiota appears to be crucial in inducing these tolerogenic functions in cDCs. Microbial signaling to cDCs via TLRs can either induce Treg responses or promote regulatory properties of cDCs themselves. Interestingly, besides priming cDCs to respond to pathogenic stimuli, commensal bacteria-induced IFN-I production also seems to be relevant in the induction of tolerogenic reactions. Bacterial, TLR-dependent signaling was shown to promote IL-27 and IL-10 production in colonic cDCs and subsequently induce pTregs in an IFN-I dependent fashion [84]. On the same subject, upon exposure to *Clostridium butyricum*, cDCs are activated to produce TGF-β, which induces the generation of pTregs [85]. Moreover, commensal bacteria, such as *Bacteroides fragilis* and *Bacteroides thetaiotaomicron*, utilize outer membrane vesicles (OMVs) to promote tolerance and anti-inflammatory properties of DCs. By either direct signaling or delivery of immunomodulatory molecules, such as PSA, they were shown to positively impact IL-10 signaling in intestinal cDCs. The TLR-2-mediated sensing of OMV-associated PSA was shown to ameliorate colitis severity in an experimental TNBS-colitis model, by increased induction of IL-10-producing Tregs, whereas cDC-derived IL-10 was elevated in a human in vitro system upon OMV exposure [86, 87]. Interestingly, these OMVs were unable to elicit the IL-10 expression in colonic cDCs derived from UC patients, pointing out possible alterations in the response of cDCs towards commensal-derived OMVs in IBD [87].

Another essential mechanism of intestinal tolerance involves plasma cells in the gut producing IgA antibodies. These antibodies extensively coat intestinal bacteria, a process shown to be crucial for regulating and maintaining the colonization of the intestinal microbiota [88]. cDCs can sample commensal bacteria through the intestinal M cell layer in Peyer’s patches and lymphoid follicles, presenting them to intestinal B cells in the mesenteric lymph nodes to induce IgA secretion, both in a T-cell-dependent and -independent manner [89]. In the Peyer’s patches, cDC2s interact with activated B cells to induce IgA class-switch recombination (CSR), which is dependent on integrin αvβ8 expression in cDCs and consequently promotes TGF-β activation during B cell interactions [88]. Interestingly, it was also shown that lung DCs can induce IgA CSR in lung B cells via MyD88-dependent TLR signaling and upregulate gut-homing markers, such as integrin α4β7 and CCR9. Consequently, they can direct the migration of IgA^+^ B cells to the gut, thereby increasing protective IgA levels against enteric pathogens after intranasal vaccination in the presence of microbiota [90].

Taken together, the microbiota can influence intestinal cDCs through PRR signaling to enhance pathogen clearance, while also providing critical signals to cDCs that promote intestinal tolerance.

### Influence of the microbiota via bacterial metabolites

In addition to direct effects of the microbiota via PRRs, commensals can also indirectly influence cDCs by encountering microbiota-derived compounds. As the bacterial load increases towards the distal intestine, so does the abundance of their metabolites [91]. Commensal-derived metabolites are the result of the bacterial metabolism of dietary components that could not be taken up by the host cells, or enzymatically-modified host-derived substances. In addition, molecules derived from bacterial homeostatic metabolism or used for bacterial signaling can interfere with immune cells in the intestine. Similar to PRR-signaling, bacterial metabolites can influence cDCs by either promoting the induction of tolerance or increasing inflammatory reactions against pathogens (Fig. 3).

Among those metabolites, short-chain-fatty-acids (SCFAs), resulting from the fermentation of dietary fibers in the colon, are described most extensively for their tolerogenic effects on the host immune system, most notably for their impact on the acetylation of the Foxp3 locus and thereby increase in colonic pTregs differentiation [92]. However, receptors for SCFAs, such as butyrate, are widely expressed on cDCs as well. Butyrate was shown to promote anti-inflammatory properties in cDCs, resulting in increased pTreg and IL-10-producing Tr1 cell differentiation, as well as elevation of sIgA levels in the lumen by T-cell-independent IgA class switch in B cells [93–96]. Some studies could show that the G-protein-coupled-receptor Gpr109a was involved in the protective effect of butyrate, both in a DSS-colitis model using Gpr109a-deficient mice and in human moDCs [95, 96]. By this butyrate-dependent conditioning of cDC function, SCFAs can increase IgA-levels in the mucus layer, as well as the frequencies of regulatory pTregs and Tr1 cells, thereby contributing to maintaining gut homeostasis.

To facilitate the uptake of nutrients, especially fatty acids, primary bile acids (BAs) are secreted into the SI, where the vast majority is reabsorbed for digestive processes. However, BAs can also be metabolized through dihydroxylation, deconjugation, or conjugation by commensal bacteria when entering the large intestine. Although the effects on intestinal cDCs of both primary and secondary BAs are not yet fully elucidated, human and murine data indicate that BAs impact cDC function and development upon exposure to these metabolites, primarily endowing them with tolerogenic properties. Several studies have shown the effect of bile acids on cDCs, primarily through the Farnesoid X receptor (FXR) or the Takeda G-protein-coupled receptor 5 (TGR5). The secondary BAs lithocholic acid (LCA) and deoxycholic acid (DCA), as well as the primary BA taurochenodeoxycholic acid (TCDCA) were shown to reduce the production of pro-inflammatory cytokines, such as IL-12, IL-1β, IL-6, and IL-23, in activated cDCs in a TGR5-dependent manner [97, 98]. Functionally, this was shown in a model of experimental autoimmune uveitis, where LCA could attenuate severity, with this effect being partially reversed in TGR5-deficient mice [98]. By antagonizing the FXR, the secondary BA isodeoxycholic acid (isoDCA) was shown to induce an anti-inflammatory state in cDCs. Exposure to the bile acid induced alterations in the expression of Ag presentation-related genes in cDCs, as well as increased DC-dependent pTreg induction in mice harboring isoDCA-producing bacterial strains [99]. However, the anti-inflammatory effect of DCA is also a topic of controversy, as DCA and chenodeoxycholic acid (CDCA)-primed BMDCs have been shown to promote the production of the pro-inflammatory cytokines IL-1α and IL-1β in vitro [100].

Other bacterial metabolites were also shown to have an impact on immune cells in the intestine, such as polyamines and tryptophan metabolites, like indole or indolic acid derivatives [101]. However, their impact on cDC function has not been thoroughly studied. In line with this, while most metabolites produced by the host or bacteria serve as signaling molecules towards human host cells, quorum sensing is a communication system mainly used by prokaryotic cells, comparable to hormones in eukaryotes. These signal molecules, also called bacterial auto inducers (AI), are small compounds used by bacteria for intraspecies, but also for interspecies communication [102]. Overall, data on how these AIs influence DCs in the intestine is scarce, yet it was shown that *Pseudomonas quinolone* signal (PQS) decreased T cell proliferation induced by BMDCs exposed to the AI in vitro, pointing towards a role of quorum sensing influence on DC-mediated T cell reactions in the intestine [103].

To conclude, commensal bacteria can prime cDCs through PRR signaling and bacterial metabolites in two ways: they can either instruct pathogen clearance and an effective adaptive immune response or promote tolerance by inducing anti-inflammatory DCs and subsequent tolerogenic immune cells.

The commensal microbiota contributes to the primary functions of intestinal conventional dendritic cells (cDCs) by training them to respond and induce an immune response against pathogens, as well as by promoting their tolerogenic properties. (a) The commensal microbiota and in particular polysaccharide A from *B. fragilis* can induce IFN-I production in plasmacytoid dendritic cells (pDCs) and cDCs themselves, leading to a systemic increase in ISG expression. Through this mechanism, cDCs can then contribute to adequate viral clearance by inducing NK and T cell responses. Similarly, during *Mycobacterium tuberculosis* infection, the microbiota, including *L. plantarum*, signals to cDCs to promote T effector memory cells and thereby effectively decreases bacterial survival. (b) In the colon, commensal bacteria including *C. butyricum* and their metabolites, such as short-chain-fatty-acids (SCFAs), bile acids, and outer membrane vesicles (OMVs) from *B. fragilis* and *B. thetaiotaomicron*, dominantly act on host immune cells and can induce regulatory T cells (Tregs) and Tr1 cells, as well as IL-10 expression by cDCs. Furthermore, class-switch recombination (CSR) and, consequently, sIgA secretion by plasma cells are promoted via cDCs. Additionally, microbiota-induced IFN-I is capable of inducing IL-23 and IL-10 expression by cDCs and consequently promoting both tolerogenic properties in cDCs, as well as Tregs.

## Changes in DCs and the microbiota along the intestine

### Specialized DC subsets in distinct intestinal locations

The cellular and functional landscape of the gut changes along the transcending axis. The upper intestine is characterized by low pH, a high abundance of nutritional Ags, and vitamins, such as vitamin A and its derivatives, and serves the physiological function of nutrient uptake. In contrast, the increased pH, lower oxygen levels, lower flow rate, and availability of fermentable carbohydrates lead to an increase of bacterial load in the colon, and consequently elevated abundance of bacterial metabolites such as SCFAs [104, 105]. These facts imply that cDCs in both locations are subject to different environmental conditions and encounter distinct sources of Ags or metabolites. Thus, one can assume that cDCs in the SI have higher chances of being influenced by food Ags and nutrients, such as glucose and fatty acids. In contrast, colonic DCs are mostly affected by commensal-derived products. Indeed, data gathered from mice and humans shows that the local microenvironment of the distinct regions of the intestine harbors distinct cDC subsets [106–108]. While the upper intestine is richer in cDC2As, the colon shows increased percentages of cDC1s [106]. Moreover, cDCs seem to have distinct characteristics depending on the location from which they come. Cd274 (PD-L1) and Cd209b expression are upregulated in cDC2s in the duodenum, whereas colonic cDCs express higher levels of Xcr1 and S100a4 [108]. Similarly, human colonic DCs express more CCR7, CCR4, and the inhibitory receptor ILT3, and display an enhanced endocytic capacity, especially in the cDC2A subset, whereas CCR9 is increased in ileal cDCs [107].

Aside from the differences in distribution, cDCs in distinct intestinal regions were also shown to have functional specializations. cDCs from the SI promote a Th2 gene signature in adoptively transferred naïve OT-II cells isolated from mice challenged with oral and intrarectal OVA, whereas colonic cDCs can induce genes related to Th17 differentiation [108]. Additionally, cDCs from both locations appear to have pTreg-polarizing capacities, although differences in the upregulated molecular pathways suggest distinct differentiation programs are engaged in each region. Indeed, adequate glucose levels in the SI determine RALDH activity in cDC2As, thereby increasing RA synthesis and promoting the differentiation of pTregs [109]. However, human colonic DCs exhibit a larger capability of inducing IL-10-producing Foxp3^+^ Tregs in vitro compared to cDCs derived from the ileum of healthy donors [107]. Thus, SI cDCs may rely on RA synthesis to promote pTregs, whereas colonic cDCs could be influenced by the elevated levels of bacterial metabolites in the distal intestine. Furthermore, Denning et al. showed that the breeding facility of the mice used for the experiments influenced the capacity of Th17 and Treg differentiation by intestinal cDCs, thereby hinting towards the importance of the intestinal microbiota on cDC function [106].

Taken together, growing evidence emerges that DCs from distinct intestinal regions differ in function and phenotype; however, the analyses so far have not delved into the molecular mechanisms that define these differences. Besides, what aspects of the local microenvironment trigger the acquisition of regional specialization by cDCs are incompletely defined.

### The longitudinal and transversal axis of the intestinal environment

The commensal microbiota and its metabolites in the human intestine undergo permanent oscillations upon changes in diet, disease, and daytime [105, 110, 111]. Yet, besides these variable factors, and in a similar manner as the cellular immune structure, the gut biogeography of the microbiota is vastly influenced in composition and density by the environmental changes along the transcending intestine [104]. Recent advances in sampling techniques allowed the acquisition of luminal content under physiological conditions, revealing changes in microbial composition, bile acids, and host metabolome along the longitudinal axis [105, 112]. Remarkably, dramatic differences were observed between the intestinal and stool metabolomes, highlighting the importance of precise sampling location in microbiota studies.

In addition to alterations in the microbiota along the longitudinal axis, the composition and environment from the lumen to the mucosa and crypts vary drastically [113]. Colonic crypts and the mucus layer form a special anatomical area protected from the fecal stream and thereby allow distinct bacteria to colonize these niches [104]. The mucus layer in the gut not only influences the bacteria that can colonize the colonic crypts, forming a “Crypt-specific Core Microbiota”, but also oral tolerance is promoted by mucus signaling to intestinal cDCs via MUC2 to induce tolerogenic cDCs and promote oral tolerance [114, 115].

To conclude, an intricate crosstalk appears to govern the local microenvironment in the intestine, whereby the intrinsic characteristics of each intestinal region influence which species of microbes can colonize and what type of immune cells, in particular cDCs and T cells, can seed there. In addition, commensals and immune cells have developed ways to interact specifically with each other and shape the immune response locally.

### Limitations of the current microbiome–cDC studies

The microbiota acts through several mechanisms on intestinal cDCs and is a well-established factor in the pathogenesis of IBD [1]. Recent multi-omics data support the notion that damage to the intestinal barrier upon inflammation enables the translocation of bacteria into the tissue, allowing direct interactions of host immune cells with bacterial Ags [11]. However, technical limitations in isolating a sufficient number of DCs from the intestine, as well as the impossibility of mimicking the microenvironment of distinct intestinal regions for in vitro studies, limit the knowledge we can obtain from both human and murine studies.

Additionally, a caveat in investigating the influence of microbiota on IBD and intestinal immune cells is that most data is derived from fecal samples, sparing the extensive differences among longitudinal and transversal locations. Distinct microbial profiles of mucosa-associated bacteria were described for ileal and colonic CD, pointing towards the importance of both the longitudinal and transversal axis of the microbiota [116]. Interestingly, analysis of the intestinal mucosa and fecal samples of a treatment-naïve pediatric CD patient cohort revealed differences in mucosal microbiome samples in comparison with non-IBD controls, without these differences being reflected in fecal microbiota samples [117]. Therefore, the widely described changes in the microbiota observed in IBD may be a secondary effect and should be interpreted carefully when discussing microbial effects on immune cells in IBD. Notably, these aspects are also largely overlooked in the context of cDCs in IBD, which confounds the actual role of these cells in IBD pathogenesis and hinders the development of novel DC-based therapies.

Overall, there is limited information about the differences within cDC populations along the intestine, which also considers the changes in the microbiota and the differences between mucosa-associated and fecal bacteria.

### cDC-T cell interactions–new methods and difficulties

Currently, several limitations exist regarding cDC studies in the intestine, as it is challenging to simulate all the conditions present in the tissue, including local factors and the microbiota. Considering that the primary functions of cDCs stem from their ability to interact with T cells, innovative methods have emerged to study cDCs and their respective interaction partners in vivo, allowing for the integration of the different intestinal dimensions and changing microbial influences. Approaches vary from sequencing-based techniques to photocatalytic proximity labeling, enzymatic labeling, and Ag tagging (Table 2). Sorting of physically interacting cells (PICs) is a sequencing-based technique, which enables the capture of strong interactions taking place at a specific, restricted time point. By choosing a surface marker for each targeted interaction partner and carefully isolating the cells, the events positive for both markers can be sorted by FACS as PICs. However, subsequent single-cell sequencing of the PICs requires computational deconvolution, and therefore, interacting cells must be transcriptionally distinct. The choice of markers used for FACS sorting is also a critical point in this strategy. Yet, this method can be easily expanded to a variety of interaction pairs [118].

Another tracking method is the LIPSTIC (“Labelling Immune Partnerships by SorTagging Intercellular Contacts”) mouse model, which labels interacting cells through an enzymatic reaction. It utilizes the *Staphylococcus aureus* enzyme transpeptidase sortase A (SrtA) to transfer a biotin tag and label the interaction between a predefined receptor-ligand pair (CD40-CD40L). In this case, CD40L is genetically fused to SrtA while its interacting partner CD40 expresses a tag of five N-terminal glycine residues (G5). For the labeling to occur, SrtA must first be loaded with a substrate containing the sorting motif, such as an LPETG peptide linked to a biotin label. When ligand and receptor interact, SrtA catalyzes the transfer of the substrate onto the G5-expressing receptor. Finally, the labeled receptors can be detected by the expression of biotin via FACS [119]. By increasing the levels of expression of both SrtA and G5 in donor and acceptor cells on the cell membrane, and driving their expression via the ROSA26 locus, the model has been recently expanded to a universal LIPSTIC model. Here, the enzymatic labeling is not restricted to a particular receptor-ligand combination but can tag any cell-cell interactions between donor and acceptor cells [120]. The LIPSTIC models have been tested in viral and tumor settings and revealed that a small fraction of hyperactivated DCs drive most of the Ag presentation to CD4^+^ T cells in the tumor microenvironment [120, 121]. These methods require prior genetic modifications of the cells. Still, they allow the capture of strong, direct cellular communications and record all interactions cumulatively from the time point of substrate injection. However, more subtle communications, e.g., via cytokine products of DCs, may be missed. Other similar available methods include EXCELL [122] and FucoID [123].

Avoiding genetic modifications in labeling and tracking systems broadens the possibilities and feasibility of use. Different chemical labeling methods, such as photocatalytic proximity labeling (PPL), have shown promise in investigating cell-cell interactions. However, naturally occurring chromophores in living systems limit the effectiveness of labeling systems using shorter wavelengths of light in vivo [124]. With CINTER-Seq, a novel pipeline was recently introduced, combining PPL with a subsequent single-cell sequencing approach [125]. It allows for labeling of interacting cells in vivo with a biotin tag, using an antibody-mediated PPL and irradiation under near-infrared (NIR) light. Conversion of the biotin label into a sequenceable DNA barcode enables the subsequent analysis of the cellular interactome at single-cell resolution. As the PPL is antibody-based, the system can be adapted to a variety of surface molecules. However, careful consideration must be taken to ensure the correct antibody concentration, choice of molecular target, and irradiation times to avoid a reduced signal-to-noise ratio.

To investigate interactions between Ag-specific T cells and APCs, the chemical labeling method Fluorescein-AsH (FlAsH) provides a fluorescence-based tool, assuming the Ag is known. No genetic modifications are required, as tracking APC-T cell interactions relies on tetracysteine tagging of the Ags, which enables the visualization of both Ag-loaded APCs and recipient T cells. Treating cells or organisms with the FlAsH probe induces fluorescence by binding to the cysteine-rich region, allowing for the detection of interacting cells through FACS or fluorescence-based intravital microscopy techniques [126]. This method facilitates the study of Ag-specific T-cell interactions and offers the opportunity to examine TCR affinity and, therefore, interaction strength, in contrast to other models. Compared to single-step methods of labeling Ags, this method achieved a reduction in background noise. However, this approach cannot be used if the Ag is unknown or if the interaction is non-Agantigen-specific.

Taken together, new methods for studying cellular networks have become available to researchers in recent years. They provide precise tools to investigate cellular communications that rely on strong receptor-ligand interactions or chemical bonds and require a very close proximity of interacting partners.

Currently, most methods depend on administering a substrate, which allows for capturing interactions at specific time points; however, this may overlook the flexible nature of cell-to-cell interactions. Additionally, they do not account for paracrine modes of cell communication, which are a major aspect of immune cell function. Therefore, there remains a need to develop methods that reflect the dynamic nature of cellular interactions in the intestine and also consider the exchange of soluble factors.

**Table 2:** Cell-cell contact labeling methods

		Mechanism of labeling	Main characteristics	Genetic modifications
Physical interaction	**PIC-Sorting** [118]	Sorting of physically interacting Cell A and Cell B doublets with subsequent scRNAseq	• Surface molecules on Cell A and Cell B must be known and Ab-tagged • Capture of strong interactions • Requires computational deconvolution & scRNAseq pipeline	no
Enzymatic labeling	**LIPSTIC** [119]	SrtA-expressing Cell A is loaded with biotin-LPETG. Upon CD40L–CD40 interaction with G5-tagged Cell B, SrtA enzymatically transfers the label to Cell B	• Restricted to CD40L-CD40 interaction • Biotin-tagging allows for various analysis methods (FACS, IF, scRNAseq) • Interaction strength quantification (Biotin count)	yes
	**Universal LIPSTIC** [120]	Similar to LIPSTIC, but driving expression of both SrtA and G5 on the cell membranes of Cell A and B via the ROSA26 locus	• The enzymatic labeling is not restricted to a particular receptor-ligand combination • Suitable for adoptive transfer experiments • Fully endogenous model available: Cre-positive cells express SrtA, Cre-negative cells express G5	yes
Chemical labeling	**FlAsH** [126]	Tetracysteine tag conjugated to an Ag emits fluorescence upon binding the FlAsH probe; Labeling of Ag-loaded Cell A, and interacting Cell B	• Relies on MHC-TCR interactions • Possible to investigate TCR affinity • Improves background labeling compared to single-step methods • Ag needs to be known and modified • Not suitable for non-Ag-specific interactions	no
	**CINTER-Seq** [125]	Ab-mediated tagging of Cell A with PPa, a porphyrin; NIR-light irradiation triggers photosensitization of the porphyrin, leading to the generation of ^1^O_2_, which can oxidize surface molecules on Cell B. Finally, the oxidized molecules on Cell B are prone to bind to a nucleophilic biotin probe	• Surface molecule of Cell A needs to be known and tagged with the Ab-PPa • Subsequent scRNAseq pipeline allows for TCR sequencing and analysis of interaction strength (Biotin quantification) • Requires laser exposition of tissue of interest • Limited tissue penetration of the laser	no

*PIC *physically interacting cells; *scRNAseq *single cell RNA sequencing; *Ab *Antibody; *LIPSTIC *Labelling Immune Partnerships by SorTagging Intercellular Contacts; *G5 *Tag with five N-terminal glycine residues; *SrtA *Sortase A; *IF *Immunofluorescence; *FlAsH *Fluorescein-AsH; *Ag *Antigen; *CINTER *cellular interactome; *PPa *Pyropheophorbide-a; *NIR *near-infrared

### cDCs in IBD management

cDCs are increasingly recognized as essential modulators of immune balance in the gut, with growing evidence highlighting their role in maintaining homeostasis and in disease development. So far, strategies to target these cells in IBD have been scarce, most Likely due to their undefined role in the pathogenesis of the disease. However, a recent study revealed that the integrin α 4β7-blocking drug Vedoluzimab mainly acts by preventing the migration of CD1c^+^ DCs to the intestinal mucosa [70]. This contradicts the initial presumption that Vedoluzimab blocks the trafficking of CD4^+^ memory T cells to the intestine and shows evidence of cDCs as key players in the pathogenesis of IBD. Furthermore, cDC2s were shown to express multiple genes associated with α-TNF resistance. They may play an underappreciated role in resistance to α-TNF therapy, as a close correlation has been found between activated cDCs, T cells, and B cells within α-TNF-resistant lesions in IBD patients [4, 5]. Oncostatin M (OSM), which is highly produced by cDC2s, is discussed as a potential mimic of TNF signaling in inflammatory monocytes, potentially contributing to therapy failure [5]. This suggests that cDCs could amplify α-TNF resistance and serve as potential targets to enhance the efficacy of α-TNF blockade. Thus, cDCs are attractive targets for future IBD therapies, though clinical studies specifically focusing on cDCs remain limited. One promising approach involves the ex vivo production and administration of tolerogenic DCs (tolDCs). For example, Phase I trials in refractory Crohn’s disease have demonstrated that intraperitoneally injected, autologous tolDCs are safe and feasible, although their large-scale effectiveness still requires validation [127]. These examples underscore the importance of understanding the cell-cell contacts of cDCs in IBD to further elucidate the potential mechanisms and pathways through which cDCs influence IBD therapy. The new labeling methods described above can help elucidate whether existing drugs, supplements, probiotics, or traditional medicines may have underestimated effects on cDCs, as seen with Vedolizumab. Ultimately, more basic and translational research is necessary to better understand the diverse roles of cDCs in IBD and to develop strategies for their therapeutic manipulation, aiming to restore intestinal immune balance and reduce dependence on conventional, broad immunosuppressants.

### Final remarks

In this review, we focus on the primary functions of cDCs in the intestine and how the microbiota influences the fine-tuning and shaping of these functions. Additionally, we explore the role of cDCs in IBD, a disease that is particularly affected by changes in the microbiota. However, the exact role of cDCs in IBD development remains unclear, with several factors contributing to this gap in knowledge. First, the unequivocal distinction of cDCs and their subtypes from other myeloid cells has only recently become possible through advances in single-cell sequencing techniques. Second, the impact of the microenvironment has only recently gained attention. It is now evident that local tissue factors and the microbiota significantly affect the phenotype and function of immune cells. Nonetheless, studies that integrate both aspects and examine their effects on cDCs and their cellular networks are limited. Finally, methods to study cell–to–cell interactions in vivo—considering the complexity of the intestinal environment—are still being developed, although new, sophisticated methods have emerged in recent years. Overall, we now have better tools to study cDC biology in the intestine and how these cells may contribute to IBD. Incorporating analysis of the microbiota and local tissue microenvironment into the crosstalk between cDCs and T cells will help us better understand their role in IBD and guide the development of new therapeutic strategies.

## Data Availability

No original data was generated for this review article, so data sharing is not applicable.
